# RETRACTION: Deciphering the Role of Wound Healing Genes in Skin Cutaneous Melanoma: Insights into Expression, Methylation, Mutations, and therapeutic Implications

**DOI:** 10.1111/iwj.70543

**Published:** 2025-04-18

## Abstract

**RETRACTION:** ZhangY. , GaoC. , LuoJ. , KhanA. , Salem‐BekhitM. M. , SalemM. M. , QiZ. , and JiangB. , “Deciphering the Role of Wound Healing Genes in Skin Cutaneous Melanoma: Insights into Expression, Methylation, Mutations, and therapeutic Implications,” International Wound Journal 21, no. 4 (2024): e14807, 10.1111/iwj.14807.38591163 PMC11002634

The above article, published online on 09 April 2024, in Wiley Online Library (http://onlinelibrary.wiley.com/), has been retracted by agreement between the journal Editor in Chief, Professor Keith Harding; and John Wiley & Sons Ltd. Following an investigation by the publisher, all parties have concluded that this article was accepted solely on the basis of a compromised peer review process. The editors have therefore decided to retract the article. The authors did not respond to our notice regarding the retraction.

**RETRACTION:** Y. Zhang , C. Gao , J. Luo , A. Khan , M. M. Salem‐Bekhit , M. M. Salem , Z. Qi , and B. Jiang , “Deciphering the Role of Wound Healing Genes in Skin Cutaneous Melanoma: Insights into Expression, Methylation, Mutations, and therapeutic Implications,” International Wound Journal 21, no. 4 (2024): e14807, 10.1111/iwj.14807.38591163 PMC11002634

The above article, published online on 09 April 2024, in Wiley Online Library (http://onlinelibrary.wiley.com/), has been retracted by agreement between the journal Editor in Chief, Professor Keith Harding; and John Wiley & Sons Ltd. Following an investigation by the publisher, all parties have concluded that this article was accepted solely on the basis of a compromised peer review process. The editors have therefore decided to retract the article. The authors did not respond to our notice regarding the retraction.

